# A Retrospective Review of Neonatal Sepsis among GBS-Colonized Women Undergoing Planned Cesarean Section after Labor Onset or Rupture of Membranes

**DOI:** 10.1155/2020/4365259

**Published:** 2020-01-16

**Authors:** Fadi B. Yahya, Matthew A. Hathcock

**Affiliations:** ^1^Mayo Clinic Health System, 404 W. Fountain St. Albert Lea, MN 56007, USA; ^2^Mayo Clinic Rochester, 200 1st Street SW, Rochester MN 55905, USA

## Abstract

**Background:**

Sepsis is a leading cause of mortality and morbidity in neonates, with group B streptococcus (GBS) remaining the most frequent pathogen isolated from term infants. Surveillance data showed that the majority of cases of early-onset GBS disease were neonates born to women who either received no or suboptimal intrapartum antibiotic prophylaxis with a notable portion of those women having a missed opportunity to receive ≥4 hours of chemoprophylaxis. Women planning delivery by cesarean section who present in labor or rupture of membranes prior to their scheduled surgery are unlikely to receive optimal GBS chemoprophylaxis and thus their neonates are at risk of having sepsis. *Materials and Methods*. A retrospective cohort study of women-infant dyads was extracted from the Consortium on Safe Labor dataset. Women who had an unlabored cesarean section at ≥37 + 0 week gestation were selected and divided into four groups based on GBS status and timing of cesarean section with respect to onset of labor or rupture of membranes. The rate of neonatal sepsis and the patterns of intrapartum antibiotic chemoprophylaxis were determined.

**Results:**

The sepsis rate (4.5%) among neonates of GBS-colonized women having their unlabored cesarean section after onset of labor or rupture of membranes was significantly higher than that in any other group in this study. In this group, 9.4% of women received chemoprophylaxis for ≥4 hours, while 31% had a missed opportunity to receive ≥4 hours of chemoprophylaxis.

**Conclusion:**

This study suggests that neonates of GBS-colonized women having a planned cesarean section after onset of labor or rupture of membranes are at increased risk of having a sepsis diagnosis. This finding suggest the need for additional studies to assess the risk of sepsis among neonates of women in this group.

## 1. Introduction

Sepsis is a leading cause of mortality and morbidity in neonates, with group B streptococcus (GBS) remaining the most frequent pathogen isolated from term infants [1]. Prevention strategies based on universal screening and intrapartum chemoprophylaxis to reduce vertical transmission of invasive GBS disease in at-risk women resulted in substantial reduction in early-onset GBS disease [2, 3]. In 2002, the Centers for Disease Control and Prevention (CDC) recommended universal antenatal screening at 35 to 37 weeks of pregnancy and intrapartum chemoprophylaxis to all GBS-colonized women at the time of labor or premature rupture of membranes (PROM) including women planning delivery by cesarean section [4]. The only exception was for GBS-colonized women who have a planned cesarean delivery prior to the onset of labor or PROM [4]. Those recommendations were then endorsed in the 2010 CDC Prevention of Perinatal Group B Streptococcal Disease guidelines; however, The American College of Obstetricians and Gynecologists (ACOG) in a recent committee opinion suggested that a single dose of an antibiotic (or combination of antibiotics) that provides GBS prophylaxis and presurgical prophylaxis is appropriate for GBS-colonized women with a planned cesarean birth who present in active labor or PROM before their scheduled delivery [5]. Intrapartum antibiotic prophylaxis (IAP) is most effective if administered at least four hours before delivery [6–8]. CDC guidelines identify asymptomatic infants born to mothers who were colonized with GBS but received <4 hours of IAP, as at-risk for sepsis [4, 9]. A recent multistate surveillance study showed that around 70% of cases of early-onset GBS disease were neonates born to women who did not receive IAP and another 15% were among women who received IAP for <4 hours [10]. Among women who had <4 hours of IAP, more than 50% were admitted to the hospital for >5 hours and would have had an opportunity to receive IAP for an adequate duration. Women planning delivery by cesarean section who present in labor or rupture of membranes prior to their scheduled surgery are unlikely to receive optimal GBS chemoprophylaxis. The objective of this study was therefore to determine the rate of neonatal sepsis among GBS-colonized women who have their planned cesarean section after onset of labor or PROM and to assess the pattern of IAP utilization and the rate of missed opportunity to receive IAP.

## 2. Methods

We derived our data from the Consortium on Safe Labor (CSL) dataset. This dataset was extracted from the medical records of 12 institutions between 2002 and 2008. Data included demographics, prenatal complications, labor and delivery information, and maternal and neonatal outcomes. The CSL cohort consisted of a total of 228,438 deliveries at ≥23 weeks gestation, with 9.5% of women (*N* = 5,053) contributing >1 birth during the specified time period. Prior publications from the CSL have described data linkage, cleaning, recording, and validation [11, 12]. We excluded three centers that did not submit data on antibiotic usage in labor (40,674). We also excluded woman who gave birth before 37 + 0 weeks of gestation (30,019). Women who did not give birth by unlabored cesarean section (151,200) were also exempt from the study. We defined unlabored cesarean section as a delivery with the following characteristics: primary or repeat cesarean section, less than or equal to two vaginal examinations before delivery, and excluding cases with induction or unknown reason as the indication for admission. We included women who contributed multiple births during the study period. The final sample size was 6,545 deliveries. In the analysis of descriptive statistics, we included only the first delivery for each woman; thus, the sample size for that analysis was 6,401 deliveries. In evaluating the primary outcome, we included all live born neonates from our final sample of deliveries with a sample size of 6,707 neonates. To calculate the neonatal sepsis rate among GBS-colonized women giving birth vaginally at term, we included all deliveries with the following characteristics: GBS-colonized women, gestational age ≥ 37 + 0 weeks of gestation, and vaginal birth. The sample size for this analysis was 27,649 live born neonates. The number of neonates with a sepsis diagnosis in this sample was 357. We divided our final sample into four groups based on the GBS status of the mother and the timing of cesarean section in relation to the onset of labor or PROM. Those groups were as follows: GBS positive and having cesarean section after onset of labor or PROM (reference group), GBS positive and having cesarean section before onset of labor or PROM, GBS negative and having cesarean section after onset of labor or PROM, and GBS negative and having cesarean section before onset of labor or PROM. We labeled the group of women who were GBS positive and had their unlabored cesarean section after onset of labor or PROM as the reference group. The following maternal characteristics were evaluated: maternal age, race, parity, cesarean section (primary vs. repeat), gestational age, and prepregnancy body mass index. The following maternal risk factors were also examined: chronic hypertension, gestational hypertension, preeclampsia, preeclampsia with severe features, superimposed preeclampsia, eclampsia, gestational diabetes, and preexisting diabetes.

The definition of unlabored cesarean section was utilized to select women whose clinical course closely resembles those who have their planned cesarean section after onset of labor or PROM. This definition is based on the fact that women who go into labor or have PROM prior to their planned cesarean delivery tend to wait for variable amounts of time before having their cesarean section. The amount of time depends on how long they wait before seeking care and then the time at the hospital for evaluation to establish a diagnosis and in preparation before their cesarean section. Thus, depending on the circumstances of the labor and delivery unit as well as the maternal and fetal status at the time of presentation, those patients are likely to wait several hours before their cesarean section is performed. The GBS status was assigned as positive or negative based on the results of the GBS screening culture. Intrapartum was defined as the period from admission to triage until delivery. IAP was defined as the initiation of a recommended antibiotic for GBS prophylaxis. Optimal chemoprophylaxis was defined as the initiation of a recommended GBS prophylaxis antibiotic for ≥4 hours before delivery [13]. Although a shorter duration of recommended IAP is less effective than ≥4 hours of prophylaxis, exposure to ≥2 hours of antibiotics has been shown to reduce GBS vaginal colony counts and decrease the frequency of a clinical neonatal sepsis diagnosis [7, 14, 15]. Thus, we defined adequate chemoprophylaxis as the initiation of a recommended GBS prophylaxis antibiotic for ≥2 hours but <4 hours before delivery.

Neonatal sepsis was the primary outcome evaluated. Sepsis rates among neonates were obtained for each of the four groups and for neonates born to GBS-positive women giving birth vaginally at term. Neonatal sepsis rates were then compared to the rate among neonates born to women in the reference group. The secondary outcomes examined were the patterns of utilization of IAP, neonatal infectious pneumonia, and neonatal intensive care unit (NICU) admission.

Maternal characteristics were summarized as means, standard deviations, and count percent as appropriate. Analysis of continuous variables was done through an ANOVA, Fisher's exact test, or Chi-square test as appropriate. Infant sepsis was analyzed using a generalized estimating equation (GEE) through logistic regression adjusting for delivery timing, before or after labor, GBS status, and the interaction between GBS status and timing of delivery. The interaction of GBS status and delivery timing is reported to address the primary endpoint. While *p* value adjustments for multiple comparisons were considered, these are not reported due rarity of neonatal sepsis. Sepsis rates among neonates of women in the reference group were compared to those of women who were GBS positive and gave birth vaginally at term using an exact binomial test. *p* values less than 0.05 were considered statistically significant. Statistical analysis was performed using SAS software version 9.4 (SAS Institute Inc., Cary, NC) and R 3.1.1 (R Foundation for Statistical Computing, Vienna, Austria).

## 3. Results

Maternal demographic characteristics are shown in (Table 1).

There was a statistically significant difference in the demographic characteristics of women who had their unlabored cesarean section before compared to after labor or PROM. Women who had their unlabored cesarean section before onset of labor or PROM were more likely to be white, multiparous, and with prior cesarean section, higher BMI, and more advanced gestational age. Those distribution differences were similar among GBS-positive and GBS-negative patients. With regard to maternal risk factors, gestational hypertension was more commonly identified in woman having their cesarean section prior to onset of labor or PROM among both GBS-positive and GBS-negative groups (Table 2).

Among GBS-negative woman, those with gestational diabetes and preexisting diabetes were more likely to have their cesarean section prior to onset of labor or PROM. Among women who were GBS positive, 18.3% had their unlabored cesarean section after onset of labor or PROM as compared to 19.6% of GBS-negative women.

We evaluated secondary outcomes among women in the reference group. In this group, there were 174 women, of which 73 (41.9%) received IAP. There were 170 women with enough information to determine the duration of IAP. Among those women, 16 (9.4%) received optimal chemoprophylaxis and 19 (11.2%) received adequate chemoprophylaxis prior to delivery. Information on the total length of stay in the hospital prior to delivery including triage time and hospital admission time was available for 138 women. Among those women, 126 were hospitalized for more than 3 hours of which 76 did not receive any IAP. Among women who did not receive IAP, 55 were hospitalized for ≥5 hours and 21 were hospitalized between 3 and 5 hours prior to delivery. Thus, for the total sample, at least 31% had a missed opportunity to receive optimal chemoprophylaxis and at least 12% had a missed opportunity to receive adequate chemoprophylaxis.

The neonatal sepsis rates were calculated for each of the four groups as shown in Table 3. The neonatal sepsis rate among GBS-positive women giving birth vaginally at term was 1.3%. Among women in the reference group, the rate of neonatal sepsis was significantly higher than the rate among GBS-positive women having their cesarean section before onset of labor or PROM (4.5% vs 1.0%, *p* < 0.01). On the other hand, the rate of sepsis was not significantly different among neonates born to GBS-negative women regardless of the timing of their cesarean section. The rate of sepsis among neonates born to women in the reference group was also significantly higher than the rate among GBS-positive women giving birth vaginally at term (4.5% vs. 1.3%, *p* < 0.01).

After adjusting for maternal characteristics (maternal age, BMI, gestational diabetes, and pregestational diabetes), neonates born to women in the reference group had a 5.32-fold (95% confidence interval (CI): 1.35-20.9, *p* = 0.01) increased risk of sepsis compared to those born to GBS-positive women who had their cesarean section before onset of labor or PROM (Table 4). Similarly, the risk of sepsis was 3.84-fold (95% confidence interval (CI): 1.22-12.04, *p* = 0.02) and 3.62-fold (95% confidence interval (CI): 1.12-11.77, *p* = 0.03) higher for neonates born to women in the reference group as compared to those born to GBS-negative women who had their cesarean section before and after onset of labor or PROM, respectively. The data also showed that neonates born to mothers who were GBS negative had a reduced risk of sepsis (odds ratio (95% CI): 0.33 (0.12-0.92); *p* = 0.03) as compared to those born to GBS-positive mothers. Similarly, neonates of women who had their cesarean section before labor or PROM had a significantly lower risk of sepsis (odds ratio (95% CI): 0.21 (0.06-0.71); *p* value: 0.01) as compared to those delivered after onset of labor or PROM, regardless of their GBS status. With regard to NICU admission and neonatal infectious pneumonia, there were no significant differences among the groups within each GBS status.

## 4. Discussion

### 4.1. Principal Findings

The rate of neonatal sepsis in the reference group was found to be statistically significantly higher than any other groups examined in this study. After adjusting for maternal characteristics, the risk of neonatal sepsis remained significantly higher in the reference group. It was also noted that neonates born to women who were GBS negative and those who had their cesarean section before labor or PROM were at significantly lower risk of sepsis. The study also found that the majority of women in the reference group did not receive optimal GBS prophylaxis. Additionally, more than 31% of women in that group had a missed opportunity to receive optimal IAP.

### 4.2. Results

The rate of neonatal sepsis among the different groups in this study was notably higher than some of the rates quoted in the literature [1, 10, 16, 17]. This is primarily related to two factors. First is the definition used to obtain the sepsis rate and second is related to how the diagnosis of sepsis was made. Defining the rate of neonatal sepsis is important and has been complicated by variation in the denominators used. When comparing rates of neonatal sepsis, it is important to note whether the denominator is comprised of the total number of livebirths or another measure [18]. The estimated incidence of term early-onset neonatal sepsis is 0.77 to 1 case per 1000 live births, with GBS remaining the most frequent pathogen in term infants [17]. The rate of early-onset GBS sepsis for term neonates is estimated at 0.23 per 1000 live births [10]. The incidence rates in those studies were calculated using case counts from the Active Bacterial Core surveillance program as the numerator and the number of live births extracted from state vital records and national vital statistics reports as the denominator [10]. As such, those surveillance studies are aimed at assessing the burden of disease in the overall population, while the aim of this study was to evaluate specific subpopulations and in this context the rates were noted to be different as expected. On the other hand, the sepsis diagnosis in this study represents a combination of probable and culture-proven sepsis [19, 20], as compared to the rates described above that were extracted from studies where the numerator includes only culture-proven sepsis which represents a small fraction of the full sepsis burden. In a neonatal sepsis case series, culture-proven sepsis typically represented approximately 5% of all clinically suspected neonatal sepsis [21]. While a portion of clinical sepsis diagnoses likely captures noninfectious syndromes such as complications of birth or metabolic instability, the limited sensitivity of blood and CSF cultures, particularly in neonates where it may be difficult to collect adequate specimen volumes and mothers may have received intrapartum antibiotics, contributes importantly to culture negative results and the lack of complete confidence in those results [16, 22]. Furthermore, a sepsis diagnosis, regardless of whether there is a true infectious process or not, exposes neonates to interventions that on the one hand are lifesaving, but on the other hand, could have negative long-term health implications such as colonization with antibiotic-resistant bacteria and perturbations of the nonresilient early-life microbiota [22]. In addition to the health implications, assessing the risk of probable sepsis is as important as culture-proven sepsis especially that it contributes to high consumption of broad spectrum antibiotics in neonatal units [22].

The high rate of neonatal sepsis in the reference group brings into question the role of preoperative antibiotic prophylaxis in the prevention of neonatal infectious morbidity. This is important as the majority of women in this group either had <4 hours of chemoprophylaxis or did not receive any IAP. The aim of preoperative prophylaxis for cesarean section is to prevent maternal infectious morbidity while minimizing the adverse effects of antibiotics on the neonate. Three reviews examined maternal and neonatal infectious morbidity in women undergoing cesarean delivery receiving preoperative prophylaxis compared with those receiving intraoperative prophylaxis after cord clamping [23–25]. There was agreement among the three reviews that preoperative administration of antibiotics leads to a significant decrease in endomyometritis and total maternal infectious morbidity. Additionally, all three reviews found no significant differences in neonatal sepsis or neonatal ICU admission. The overall conclusion was that preoperative prophylaxis likely has no impact on neonatal infectious morbidity. Cefazolin was the antibiotic predominantly used among all the trials. Some trials did not control for intrapartum antibiotic usage and there was inconsistent reporting of neonatal outcomes among the trials evaluated. Despite the limitations, the quality of evidence with regard to neonatal outcomes was described as moderate [25]. Those studies provide a general assessment of the impact of preoperative antibiotics on neonatal infectious morbidity; and thus, the findings might not be applicable to all subgroups of patients. Furthermore, cefazolin has been shown to reach its minimal inhibitory concentration (MIC) for GBS in the fetal blood within 30 minutes of administration to the mother [26], while ampicillin reaches its MIC in cord blood within 5 minutes [27]. Although this characteristic is desirable in terms of limiting neonatal exposure to antibiotics, it can also be a factor in decreasing cefazolin's efficacy for IAP when administered in the setting of preoperative prophylaxis. The success of preoperative prophylaxis is time dependent with one study showing a fourfold increase in surgical site infection when antibiotics were administered ≥2 hours prior to skin incision [28]. The current ACOG recommendation is to administer antibiotics within 60 minutes before skin incision to ensure adequate drug tissue levels [29]. On the other hand, the efficacy of IAP in the prevention of neonatal infectious disease is also dependent on the length of time; it is administered before birth. In a study examining the relationship between ampicillin timing and the rate of neonatal GBS transmission, there was a 16-fold higher rate of GBS colonization among neonates of women who had ampicillin for <1 hour as compared to >2 hours [8]. In another study, the efficacy of IAP in the prevention of early-onset GBS disease was noted to decrease by 60% when administered for <2 hours as compared to ≥4 hours prior to delivery [6]. In a third study, there was a fourfold increase in the diagnosis of clinical neonatal sepsis among GBS-colonized woman receiving IAP for <2 hours as compared to those who received antibiotics for ≥4 hours (1.6% vs 0.4%) [7]. Thus, the efficacies of both IAP and preoperative prophylaxis are time dependent; however, they are inversely related. Preoperative prophylaxis is more effective when administered closer to the time of delivery while IAP loses its efficacy the closer it is administered to the time of delivery. The available evidence suggests that the use of preoperative prophylaxis that is appropriate for GBS prophylaxis in the setting of maternal positive GBS status is of limited efficacy as compared to IAP administered for ≥4 hours and its role in the prevention of neonatal infectious morbidity in this setting needs further evaluation.

### 4.3. Clinical Implications

This study identifies a subpopulation of pregnant women whose neonates are at increased risk of having a sepsis diagnosis. The clinical course of women in this subpopulation resembles that of women who are GBS positive having a planned cesarean delivery after onset of labor or PROM. Surveillance data showed that there was high adherence to the 2002 CDC guidelines during the study period and chemoprophylaxis was administered to 87.0% of women who were positive for GBS and delivered at term [22]. Although the 2002 CDC guidelines recommended administering IAP to pregnant women who present in labor or PROM before their planned cesarean section, this study showed that the utilization of IAP was more than 50% lower for this group as compared to the overall utilization rate during the study period which could be related to the guidelines stating that a cesarean delivery need not be delayed to achieve optimal GBS chemoprophylaxis. Perhaps the low utilization of IAP in this group was one of the factors contributing to the increased risk of neonatal sepsis, and in this context findings from this study are consistent with CDC guidance identifying infants born to GBS-colonized women receiving <4 hours of IAP, as at-risk for sepsis [4, 9]. With the recent ACOG guidance [5], it is likely that the practice will shift away from administering IAP to those women regardless of the length of time they are hospitalized prior to their cesarean delivery. Thus, labor and delivery units might benefit from monitoring the rate of sepsis among neonates born to women in this group to determine their level of risk as compared to the risk among their overall term neonatal population.

### 4.4. Strengths and Limitations

The advantage of this dataset is the inclusion of a large number of women from a set of hospitals that were representative of the national population characteristics of the delivering mothers and their neonates. This study is the first to evaluate neonatal sepsis rate among women having an unlabored cesarean section after onset of labor or PROM. There are limitations to this study primarily inherent to retrospective chart review and secondary data analysis. The diagnosis of neonatal sepsis was based on a combination of ICD9 codes and chart data; thus, we could not determine the culture status of those neonates. The lack of a uniform consensus definition for neonatal sepsis, leads to variation in diagnosis and management [19]. This is a challenge of the multi-institutional nature of the dataset, where a variety of diagnostic criteria may have been used for the diagnosis of neonatal sepsis. Additionally, we could not determine the type and timing of the antibiotics used for preoperative prophylaxis. The criteria used to define an unlabored cesarean section in this study were similar to those used in other studies based on the CSL dataset [30]. There were two limitations to using unlabored cesarean section as the basis for selecting a study population that resembles women planning delivery by cesarean section. The first limitation is inclusion of women who were admitted in labor or PROM and allowed to have a brief trial of labor before a cesarean section was performed for an obstetrical indication. We believe that this scenario was infrequent; otherwise, we would have expected to see a higher IAP utilization as the utilization rate during the study period was 87%. The other limitation exclusive to the groups labeled as having their cesarean section before onset of labor or PROM was the inclusion of women who were admitted for a maternal or fetal indication but then were induced for a short period of time before they had a cesarean section. The impact of including women who were induced in the group labeled as having cesarean section before labor or PROM would be to increase the rate of neonatal sepsis in this group and thus decrease the difference in this outcome among the groups. Another limitation in our study population is the missing data for some variables. We could not assess the frequency of chorioamnionitis or endometritis because of the significant number of deliveries with missing information on those two variables in our sample.

### 4.5. Research Implications

Additional studies are needed to evaluate the sepsis rate among neonates of GBS-positive women having their planned cesarean section after onset of labor or PROM to determine whether the findings from this study are reproducible in a more contemporary obstetric population. The efficacy of a single dose of preoperative prophylaxis that is also appropriate for GBS prophylaxis in the prevention of neonatal infectious morbidity among neonates of women in this group will need further assessment especially that the available evidence suggest that preoperative prophylaxis has a limited role in the prevention of neonatal infectious morbidity.

## 5. Conclusions

Findings from this study suggest that neonates born to women who are GBS colonized and having a planned cesarean section after onset of labor or PROM are at increased risk of being diagnosed with sepsis. One of the factors possibly contributing to this increased risk is the pattern of utilization of IAP. This study showed that the majority of women in this group did not receive optimal GBS prophylaxis at a time where the CDC guidance recommended IAP for those women prior to their planned cesarean delivery when medically appropriate. With the most recent ACOG guidance, it is likely that women in this group will no longer receive IAP and instead will receive a single dose of preoperative antibiotic prophylaxis regardless of the length of time they are hospitalized prior to their cesarean delivery. Additional studies are needed to assess the rate of neonatal sepsis specifically among GBS-colonized women undergoing a planned cesarean delivery after onset of labor or PROM to see whether the findings from this study are reproducible in a more contemporary obstetric population. In view of the available evidence suggesting that the principles of preoperative prophylaxis and IAP are inversely related, such that optimizing preoperative prophylaxis compromises IAP, it is important to further evaluate the role of preoperative prophylaxis in the prevention of neonatal infectious morbidity among women in this group.

## Figures and Tables

**Table 1:** Maternal demographic characteristics.

Demographics	GBS positive		GBS negative		*p* value
	C-section after labor or PROM (*N* = 174)	C-section before labor or PROM (*N* = 769)	C-section after labor or PROM (*N* = 1,088)	C-section before labor or PROM (*N* = 4,370)	
Age (y)^a^	29.7 (6.2)	29.3 (5.6)	29.5 (6.2)	29.4 (5.3)	0.67^1^
Race^b,c^					<0.01^2^
White	65 (37.4%)	553 (71.9%)^3^	441 (40.5%)	3,029 (69.3%)^3^	
Black	49 (28.2%)	77 (10.0%)	273 (25.1%)	317 (7.3%)	
Hispanic	39 (22.4%)	94 (12.2%)	262 (24.1%)	706 (16.2%)	
Asian	13 (7.5%)	19 (2.5%)	71 (6.5%)	113 (2.6%)	
Other	1 (0.6%)	2 (0.3%)	18 (1.7%)	40 (0.9%)	
Unknown	7 (4.0%)	24 (3.1%)	23 (2.1%)	165 (3.8%)	
BMI conception^a^	26.8 (7.5)	28.4 (7.4)^3^	25.7 (6.6)	27.5 (7.3) ^3^	<0.01^1^
>1 parity^b^	126 (72.4%)	626 (81.4%)^3^	808 (74.3%)	3,697 (84.6%)^3^	<0.01^2^
Prior cesarean^b^	94 (54.0%)	506 (65.8%)^3^	609 (56.0%)	3,184 (72.9%)^3^	<0.01^2^
Gestational age^a^	38.4 (1.1)	38.7 (0.9)^3^	38.2 (1.1)	38.6 (0.9)^3^	<0.01^1^

^a^Continuous variables report means (standard deviation); ^b^categorical variables report frequency (percentage); ^c^race analyzed as white compared to nonwhite. ^1^ANOVA analysis. ^2^Chi-square test. ^3^Significant difference within GBS status.

**Table 2:** Maternal risk factors.

Risk factors	GBS positive		GBS negative		*p* value
	C-section after labor or PROM (*N* = 174)	C-section before labor or PROM (*N* = 769)	C-section after labor or PROM (*N* = 1,088)	C-section before labor or PROM (*N* = 4,370)	
Chronic hypertension	1 (0.6%)	17 (2.2%)	20 (1.8%)	113 (2.6%)	0.19^1^
Gestational hypertension	0 (0.0%)	18 (2.3%)	16 (1.5%)	135 (3.1%)	<0.01^1^
Preeclampsia & preeclampsia with severe features	6 (3.4%)	26 (3.4%)	28 (2.6%)	131 (3.0%)	0.75^2^
Superimposed preeclampsia	0 (0.0%)	4 (0.5%)	4 (0.4%)	17 (0.4%)	0.79^1^
Eclampsia	0 (0.0%)	0 (0.0%)	0 (0.0%)	1 (0.0%)	
Gestational diabetes	7 (6.0%)	44 (5.8%)	18 (2.4%)	257 (6.0%)^3^	<0.01^2^
Preexisting diabetes	10 (5.7%)	31 (4.0%)	46 (4.2%)	115 (2.6%)^3^	<0.01^2^

Categorical variables report frequency (percentage). ^3^Significant difference within GBS status. ^1^Fisher's exact test. ^2^Chi-square test.

**Table 3:** Neonatal outcomes.

Outcomes	GBS positive		GBS negative		*p* value
	C-section after labor or PROM (*N* = 179)	C-section before labor or PROM (*N* = 808)	C-section after labor or PROM (*N* = 1,128)	C-section before labor or PROM (*N* = 4,592)	
Sepsis	8 (4.5%)	8 (1.0%)^3^	14 (1.2%)	46 (1.0%)	<0.01^1^
Pneumonia	1 (0.6%)	2 (0.2%)	3 (0.3%)	26 (0.6%)	0.44^2^
NICU	21 (11.7%)	70 (8.7%)	124 (11.0%)	451 (9.8%)	0.30^1^

^1^Chi-square test. ^2^Fisher's exact test. ^3^Significant difference within GBS status.

**Table 4:** Adjusted model for neonatal sepsis outcomes.

Comparison group	GBS positive delivered after labor or PROM (odds ratio (95% CI))	*p* value
GBS positive delivered before labor or PROM	5.32 (1.35, 20.9)	0.01
GBS negative delivered after labor or PROM	3.62 (1.12, 11.77)	0.03
GBS negative delivered before labor or PROM	3.84 (1.22, 12.04)	0.02

Multivariable logistic model adjusted for age, BMI, gestational diabetes, and preexisting diabetes.

## Data Availability

The data included in this paper were obtained from the Consortium on Safe Labor, supported by the Intramural Research Program of the Eunice Kennedy Shriver National Institute of Child Health and Human Development, National Institutes of Health, through Contract No. HHSN267200603425C.
